# Current status and advances in adipose-derived stem cells therapy for diabetes mellitus erectile dysfunction

**DOI:** 10.3389/fmed.2025.1634521

**Published:** 2025-07-18

**Authors:** Jingbang Liu, Tao Jiang

**Affiliations:** The Second Hospital of Dalian Medical University, Dalian, China

**Keywords:** adipose-derived stem cells, diabetic mellitus erectile dysfunction, vascular regeneration, nerve repair, phenotypic transformation

## Abstract

Diabetes mellitus erectile dysfunction (DMED) is a severe complication highly prevalent among male diabetic patients, with a global prevalence exceeding 50%, while current therapies exhibit limited efficacy. Adipose-derived stem cells (ADSCs) have emerged as a research focus for DMED treatment due to their accessibility, multipotent differentiation potential and paracrine properties. This article systematically reviews the mechanisms of ADSCs in treating DMED: ADSCs improve cavernous vascularization and endothelial function, inhibit fibrosis and increase smooth muscle content, ameliorate cavernous neuropathy, alleviate programmed cell death in cavernous tissues and reverse phenotypic transformation of cavernous smooth muscle. Additionally, clinical studies on ADSCs therapy for DMED are summarized, along with methods to enhance the efficacy of ADSCs treatment for ED. However, further researches on the underlying mechanisms and clinical trials are needed to advance ADSC from basic research to precision medicine.

## Introduction

According to the authoritative definition established at the Fourth International Consultation on Sexual Medicine, Erectile Dysfunction (ED) referred to the persistent or recurrent inability to achieve or maintain sufficient penile erection for satisfactory sexual intercourse (1). In 2015, the global number of adults aged 20–79 with diabetes reached 415 million (approximately 1 in 11 people), of which 90% were cases of type 2 diabetes (T2DM). This figure was projected to rise to 642 million by 2040. China, as a lower-middle-income country undergoing economic transition, was among the regions with the fastest-growing prevalence (2). Patients with diabetes were more prone to developing ED, which significantly impacted male health. Currently, various treatment strategies existed to improve erectile function, such as oral medications (PDE5 inhibitors), urethral suppositories, intracavernosal injections, vacuum erection devices, penile prosthesis implantation and stem cell therapy (3). Adipose tissue was one of the most prominent mesenchymal stem cell (MSC) sources for treating ED. ADSCs were metabolically active cells that played a crucial role in vascular regeneration of damaged tissues, inhibition of apoptosis and immunomodulation. Similar to bone marrow-derived mesenchymal stem cells (BMSCs), ADSCs possessed unique self-renewal and multipotent differentiation capabilities, but their advantage lied in easier accessibility and higher yield (4).

Research indicated that DMED was prevalent among men with type 1 diabetes, type 2 diabetes and mixed diabetes phenotypes, with an overall prevalence rate of 52.5% (5). Among male patients with diabetes, the incidence of DMED was significantly higher than in non-diabetic men, nearly three times that of the latter. Furthermore, DMED patients experienced more severe symptoms and exhibited a faster progression of the condition (6). Given the high prevalence and clinical challenges of DMED, exploring novel therapeutic strategies such as ADSCs therapy was of significant importance. This article reviewed the current status and advancements in ADSCs treatment for DMED.

## Mechanisms of ADSCs therapy for DMED

### Improving vascular formation and endothelial function in corpus cavernosum

The corpus cavernosum of the penis had a sinusoidal structure that shared many similarities with the vascular system, making it a specialized vascular-like tissue. The pathophysiology of ED and cardiovascular disease (CVD) were closely interrelated. Endothelial dysfunction occurred in the early stages of both ED and CVD, disrupting the homeostatic mechanisms responsible for regulating smooth muscle contraction and vascular tone (7).

Hyperglycemia was a significant factor in cardiovascular system damage, acting through multiple mechanisms such as activation of protein kinase C (PKC), the polyol pathway, the hexosamine pathway and the formation of advanced glycation end products (AGEs). Hyperglycemia-induced oxidative stress led to endothelial dysfunction, which played a central role in the pathogenesis of both microvascular and macrovascular diseases (8). This included ED. However, the mechanism by which diabetes caused endothelial injury was quite complex. In general, hyperglycemia in diabetic patients could induce endothelial cell dysfunction, leading to reduced synthesis and utilization of nitric oxide, thereby promoting the onset and progression of DMED. ADSCs may reverse diabetes-induced endothelial damage by secreting various cellular growth factors that improved the cavernous microenvironment.

**Figure 1:**
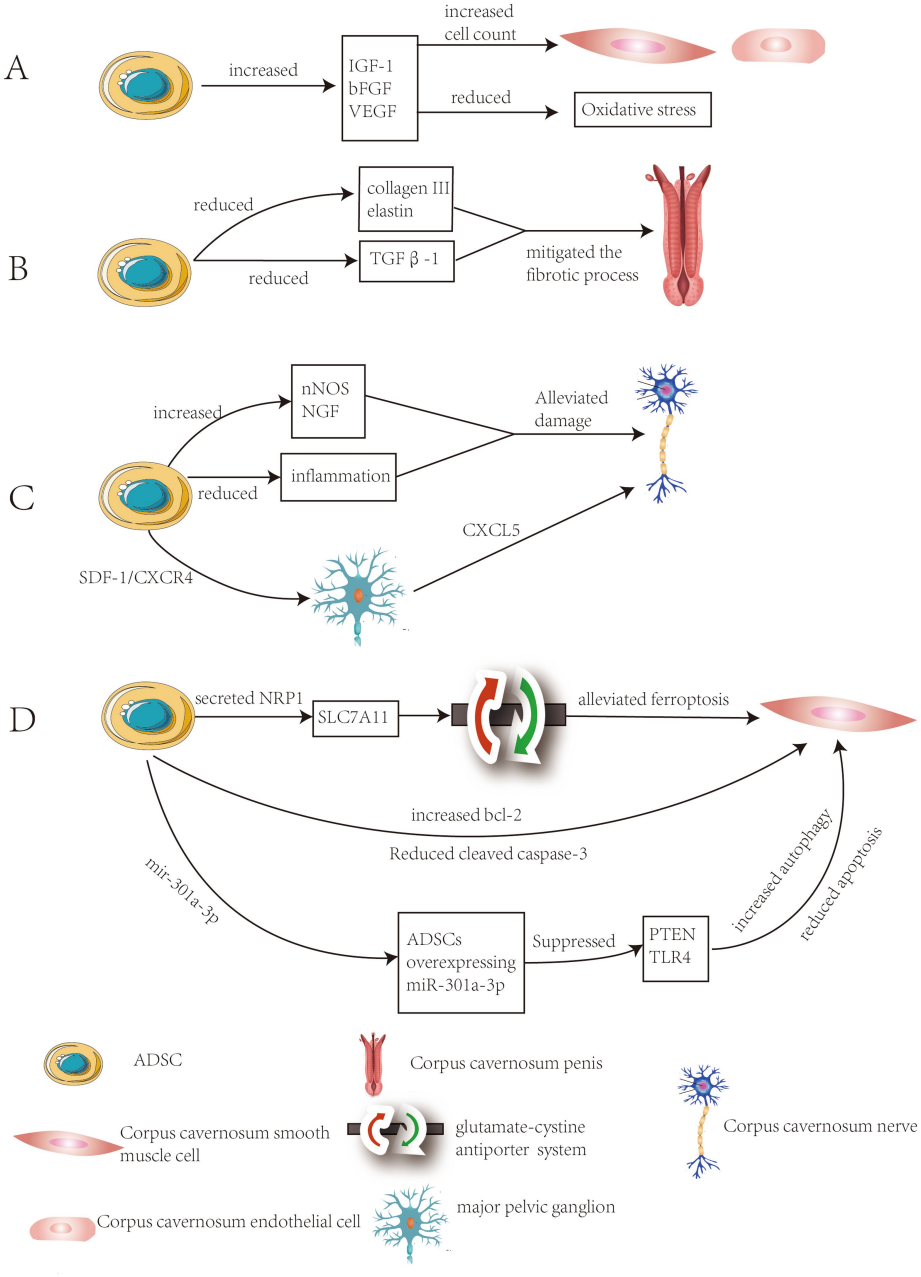
Summary of the mechanisms of ADSCs in treating DMED. **(A)** Improvement of vascular formation and endothelial function in the cavernous body. **(B)** Inhibition of fibrosis of the corpus cavernosum and increase smooth muscle content. **(C)** Improvement of neuropathy of the corpus cavernosum. **(D)** Alleviation of programmed cell death in spongy tissue cells.

Research by J. Yang et al. demonstrated that ADSCs significantly increased the concentration of insulin-like growth factor-1 (IGF-1), basic fibroblast growth factor (bFGF) and vascular endothelial growth factor (VEGF) in penile tissue through paracrine action. This enhancement elevated the content of cavernous smooth muscle and endothelial cells while reducing oxidative stress levels, ultimately improving erectile function (9). Qiyun Yang et al. investigated the therapeutic effects of combined transplantation of ADSCs and endothelial progenitor cells (EPCs) in a DMED rat model. The experimental results demonstrated that the combined transplantation improved erectile function by enhancing the expression of the endothelial marker CD31 and restoring the eNOS-cGMP-NO signaling pathway. Additionally, ADSCs promoted the proliferation and differentiation of EPCs through the secretion of vascular endothelial growth factor (VEGF) and stromal cell-derived factor-1 (SDF-1) (Figure 1A) (9).

### Inhibiting fibrosis of the corpus cavernosum and increase smooth muscle content

Chronic hyperglycemia caused by diabetes can lead to tissue damage and the development of fibrosis, thereby affecting multiple organs. A significant proportion of diabetic patients developed fibroproliferative complications, particularly Peyronie’s disease (10). Peyronie’s disease (PD) was a localized fibrotic disorder of the tunica albuginea, one of its primary characteristics being the induction of ED (11). Hyperglycemia, as the initial pathogenic factor in DMED, directly damaged the endothelial cells of the penile corpus cavernosum and arteries, triggering local inflammatory responses and the release of pro-fibrotic factors such as TGF-*β*. As a core mediator of fibrosis, TGF-β synergistically promoted vascular smooth muscle cell apoptosis and excessive extracellular matrix deposition through both Smad-dependent pathways (RhoA-ROCK, Wnt10b) and non-Smad pathways. This ultimately lead to fibrosis of the corpus cavernosum (12). Fibrosis of the corpora cavernosa reduced its elasticity and compliance, impairing effective engorgement of the sinusoids. Concurrently, the weakened ability to compress subtunical venules lead to blood leakage during erection, resulting in insufficient penile rigidity and the onset of ED (13).

The study by Fabio Castiglione et al. demonstrated that ADSCs therapy significantly reduced abnormal deposition of collagen III and elastin, prevented fibrosis in the tunica albuginea and corpus cavernosum, while preserving the normal structure of penile tissue (14). The study by Safendra Siregar et al. demonstrated that *in vivo* ADSCs injection mitigated the fibrotic process in a priapism model. This effect may be attributed to the potential of ADSCs to secrete various growth factors, which suppressed TGFβ1 and collagen production (Figure 1B) (15). The study by Wenjia Deng et al. employed a multi-omics approach to investigate the association between DMED and fibrosis, identifying 45 differentially expressed fibrosis-related genes (FRGs). The researchers pinpointed TIMP1, BMP7, POSTN as core genetic biomarkers. These genes were closely linked to diabetic complication signaling pathways and extracellular matrix-receptor interactions (16).

### Improving neuropathy of the corpus cavernosum

The autonomic nervous system regulated multiple organ systems in the body, including the cardiovascular, gastrointestinal and genitourinary systems. Chronic hyperglycemia associated with diabetes is a primary cause of small nerve fiber damage, leading to diabetic autonomic neuropathy (DAN). DAN was a subtype of peripheral polyneuropathy accompanying diabetes, one of whose clinical manifestations was ED (17). The incidence of peripheral and autonomic neuropathy was significantly higher in DMED patients compared to male diabetic patients with normal erectile function (18). In diabetic males, degeneration of the cavernous nerve (CN) was frequently observed, suggesting that DAN may critically contribute to the pathogenesis of this condition (19).

The study by Selim Cellek et al. first revealed a biphasic degeneration process of nitrergic nerves around cerebral arteries in streptozotocin-induced diabetic rats. In the first phase, nerve fibers remained intact but neuronal nitric oxide synthase (nNOS) levels decreased, a stage that could be reversed by insulin treatment; in the second phase, nitrergic neurons in the ganglia were irreversibly lost through apoptosis (20). Research by G. N. Yin et al. revealed that diabetes impairs erectile-related nerve function through multiple mechanisms: a high-glucose environment significantly reduced neurite outgrowth in the major pelvic ganglion (MPG), decreased the expression of βIII-tubulin and neurofilament proteins, and diminishes nNOS-positive nerve fibers. This disrupted nitric oxide (NO)-mediated vasodilation signaling, ultimately leading to impaired cavernous nerve signal transmission (21).

ADSCs significantly improved diabetic rats’ nerve damage through multiple pathways, including enhancing nNOS expression, secreting neurotrophic factors (such as NGF), and suppressing inflammation (22). ADSCs significantly promoted nerve repair through paracrine mechanisms in DMED. Studies had shown that ADSCs transplantation significantly upregulated the expression of nNOS in the dorsal penile nerve, and nNOS serves as a key neurotransmitter regulating erectile function (23). Following cavernous nerve injury, stromal cell-derived factor-1 (SDF-1) expression was significantly upregulated in the major pelvic ganglion (MPG). ADSCs migrated directionally to the MPG via the SDF-1/CXCR4 axis and released neurotrophic factors (such as CXCL5) through paracrine mechanisms. This promotes the survival of injured neurons and axonal regeneration while improving local tissue structure (Figure 1C) (24).

### Alleviating programmed cell death in corpus cavernosum

Dysregulated programmed cell death (PCD), including apoptosis, autophagy, pyroptosis and ferroptosis, played a pivotal role in the pathogenesis of DMED. Although distinct PCD pathways exhibited different characteristics, they were interconnected through mutual enhancement, conversion and inhibition. Abnormal activation of PCD lead to dysfunction and loss of key cells in the corpus cavernosum, triggering a secondary inflammatory cascade that ultimately contributed to the development of ED (25).

Hyperglycemia induced apoptosis of endothelial cells and smooth muscle cells in the cavernous body through oxidative stress and advanced glycation end-products activating both the mitochondrial pathway (Bax/Bcl-2 imbalance, upregulation of caspase-3/9) and the death receptor pathway (Fas/FasL) (26–28). A high-glucose environment triggered excessive autophagy through mTOR inhibition and Beclin-1 upregulation, synergizing with apoptosis to exacerbate cellular damage (29, 30). Hyperglycemia triggered ferroptosis through GPX4 downregulation, iron metabolism dysregulation (NCOA4-mediated ferritinophagy), and lipid peroxidation (excessive PUFA oxidation), leading to corpus cavernosum smooth muscle cell damage (31–33).

The experiments by Jun-qi Luo et al. demonstrated that ADSCs alleviated ferroptosis stress in corporal cavernosal smooth muscle cells (CCSMCs) and restore erectile function by enhancing the glutamate-cystine antiporter system through the interaction between secreted neuropilin-1 (NRP1) and solute carrier family 7 member 11 (SLC7A11) (34). Fengzhi Chen et al. found that after injecting ADSCs, the expression of the anti-apoptotic protein Bcl-2 in cavernous smooth muscle was upregulated, while the expression of the apoptotic protein cleaved caspase-3 and the apoptosis levels of endothelial cells and smooth muscle cells were significantly reduced compared to the phosphate-buffered saline treatment group (35). The experimental results of Li Liang et al. demonstrated that ADSCs overexpressing miR-301a-3p significantly restored erectile function in rats by promoting autophagy and inhibiting apoptosis through targeted suppression of the PTEN and TLR4 signaling pathways(Figure 1D) (36).

### Reversing the phenotypic transformation of corpus cavernosum smooth muscle

Shuhua He investigated the effects of calcitonin gene-related peptide (CGRP) on the phenotypic transformation of CCSMCs in DMED rats as early as 2011 (37). The phenotypic transformation of CCSMCs was typically analogized to that of vascular smooth muscle cells (VSMC), as current research on VSMC phenotypic transformation significantly surpassed that on CCSMC in both breadth and depth. Zhao Fan once defined CCSMC as follows: in various ED rat models or after direct external stimulation on CCSMC, the phenotype of CCSMC exhibited a shift from the “contractile type” toward the “synthetic type” or “proliferative type” (38).

In recent years, scholars had conducted preliminary explorations into the phenotypic transformation of CCSMCs under diabetic conditions. Research by Jing Zhang et al. revealed that mesenchymal stem cell-derived extracellular vesicles (MSC-EVs) delivered miR-200a-3p to suppress Keap1 expression, thereby activating the Nrf2/HO-1 antioxidant pathway, mitigating oxidative stress and ultimately reversing the phenotypic transformation in CCSMCs (39). Keming Chen et al.’s study demonstrated that Nesfatin-1 significantly improved body weight, blood glucose levels and erectile function in mice, while activating the PI3K/AKT/mTOR signaling pathway and promoting the transformation of CCSMCs toward a contractile phenotype (40).

Currently, there is no research confirming whether ADSCs treat ED by modulating phenotypic transformation of corpus cavernosum smooth muscle. However, our research group observed that after intracavernous injection of ADSCs in DMED rats, the expression of *α*-SMA in the corpus cavernosum significantly increased, while osteopontin (OPN) expression decreased. This suggests that ADSC may improve erectile functionby regulating smooth muscle phenotypic transformation, though the exact mechanism requires further exploration.

## Clinical application and challenges of ADSCs in the treatment of DMED

Research on ADSCs in DMED rat models had become increasingly well-established, yet clinical translation remained in its early stages, with limited human trials available.

As early as 2014, Garber Miguel Guillermo conducted a study involving intracavernosal injection of 1.5 × 10^7^ ADSCs in six patients with DMED. The results demonstrated that ADSCs therapy significantly improved morning erections and erection hardness. Some patients were able to achieve successful intercourse when combined with PDE5 inhibitors and a reduction in blood glucose levels was also observed. Follow-up after treatment showed no significant adverse reactions (41).

Mikkel Fode investigated the feasibility and safety of a minimally invasive same-day injection of autologous ADSCs for treating ED. A prospective case analysis of 10 men (including 3 DMED patients) with International Index of Erectile Function-Erectile Function (IIEF-EF) scores <17 demonstrated significant improvements in IIEF-EF scores at 1, 2 and 3 months post-treatment. The study utilized a dose of 4 mL of adipose tissue extract containing ADSCs, with the exact cell count not explicitly quantified. Only one patient reported mild ecchymosis at the fat harvesting site (which resolved spontaneously), with no other local or systemic adverse effects observed (42).

Currently, there are relatively few clinical studies on ADSCs therapy for DMED, and no consensus has been established regarding the optimal dosage of ADSCs. However, the safety of ADSCs is still widely recognized. We previously measured the concentration of the ADSCs we cultured, finding approximately 20 × 10^4^ cells per 10 microliters of cell suspension. Therefore, the volume of ADSCs suspension injected each time is approximately 10 milliliters. The key clinical studies were summarized in Table 1.

**Table 1:** Clinical application and challenges of ADSC in the treatment of DMED.

Year	Author	Cell type	Outcome	Dose	Safety
2014	Garber Miguel Guillermo	ADSCs	4/6 patients regained morning erections within 2 months; 5/6 achieved penetration with PDE5 inhibitors; Improved IIEF-5 scores; Reduced blood glucose levels in 5/6 patients.	1.5 × 10^7^ ASCs injected into corpus cavernosum.	Yes, no adverse effects reported (no immunosuppression needed).
2023	Mikkel Fode	ADSCs	Statistically significant improvements in IIEF-EF scores at 1, 2, and 3 months; 3/10 patients achieved minimal clinically important difference.	4 mL injected into corpus cavernosum.	Yes, only minor adverse events (e.g., blue discoloration at harvest site).

## DMED rat model

The current mainstream animal models for diabetes research are rodents, including rats and mice. Other large animals such as dogs, pigs, monkeys and rabbits have largely been phased out due to high costs, operational complexity, ethical restrictions and lengthy study durations (43).

Currently, all relevant studies on ADSCs therapy for DMED have used rats. The reasons are twofold: mice are insensitive to streptozotocin (STZ) (44), and rats may offer advantages in both corpus cavernosum pressure testing and intracavernosal injection.

### Type 1 DMED

Male Sprague–Dawley (SD) rats aged 7 weeks were intraperitoneally injected with STZ dissolved in citrate buffer (pH 4.5). Fasting blood glucose levels were measured 3 and 7 days post-injection. Rats with blood glucose levels >16.7 mmol/L on both occasions were defined as type 1 diabetes mellitus (T1DM) models (45).

### Type 2 DMED

Male Zucker Diabetic Fatty (ZDF) rats (a genetically obesity-prone strain) were selected and fed a high-fat diet (Purina 5,008) for 4 weeks to induce insulin resistance and obesity. Fasting blood glucose was measured at 3 and 7 days after feeding. Rats with blood glucose levels >16.7 mmol/L on both occasions were defined as T2DM models (46).

### Validation of DMED

Rats with ED were screened via the apomorphine (APO) test. Dissolved apomorphine hydrochloride (APO) (80 μg/kg) in physiological saline containing 100 μg ascorbic acid (1 mL/kg). Injected the APO solution into the dorsal neck of the rat. Continuously monitored the rat for 30 min post-injection. Recorded the number of penile erections and their duration. Yawning behavior may serve as a supplementary evaluation metric. Rats exhibiting no erectile behavior within 30 min were defined as DMED models.

## Methods to enhance the therapeutic efficacy of ADSCs for ED treatment

As summarized in Table 2, current strategies to enhance ADSCs efficacy include ADRCs, exosomes, genetic modification, and combination therapies, each with distinct mechanisms and outcomes.

**Table 2:** Methods to enhance the therapeutic efficacy of ADSCs for ED treatment.

Method	Core mechanism/advantage	Research model	Key outcomes
ADRCs	Retain native cellular microenvironment interactions; closed-system rapid isolation enable immediate clinical use.	Postprostatectomy ED patients.	Well-tolerated, no serious adverse events. 53% of continent patients showed significant improvement in erectile function (increased IIEF-5 scores). Short-term efficacy observed, particularly in non-incontinent patients.
ADSCs-exosomes	Carry functional proteins/nucleic acids; mediate paracrine intercellular communication	T2DM rats. BCNI rats.	Inhibit apoptosis of cavernosal endothelial cells and smooth muscle cells, improving erectile function. Significant increase in ICP/MAP. Efficacy comparable to direct ADSCs injection.
Genetically modified ADSCs	Overexpression/knockdown of target genes enhances therapeutic factor secretion.	DMED rats.	RXFP1 overexpression: Significant improvement in erectile function. miR-423-5p knockdown: Upregulated expression of eNOS and VEGFA. NLRP3 downregulation: Enhanced endothelial function and smooth muscle repair. siPDE5 modification: Increased IGF-1 and VEGF secretion, accelerated tissue recovery.
Combination therapy	Synergistic enhancement of ADSCs efficacy.	DMED rats ED rats.	LIPUS + ADSCs: Increased VEGF secretion via Piezo/ERK pathway activation. Icariin + ADSCs: Elevated ICP/MAP values, improved ADSCs survival rate.

### Adipose-derived regenerative cells (ADRCs)

Although ADSCs demonstrated therapeutic potential for ED, their reliance on *in vitro* expansion results in prolonged preparation cycles and challenges in standardization. In contrast, ADRCs, as an uncultured heterogeneous cell population, retained the advantages of native cellular interactions within their microenvironment. Moreover, ADRCs could be rapidly isolated via a closed system, offering potential for immediate clinical application.

Martha Kirstine Haahr et al. evaluated the safety and efficacy of a single injection of autologous ADRCs in patients with ED following prostatectomy. The results showed that ADRCs were well-tolerated with no serious adverse events. Among the 17 patients, 8 (all of whom had normal urinary continence) demonstrated significant improvement in erectile function, with a marked increase in IIEF-5 scores (47).

Martha Kirstine Haahr investigated the safety and efficacy of autologous ADRCs intracavernosal injection for treating ED following radical prostatectomy. Twenty-one patients received a single injection and were followed for 12 months. Results demonstrated favorable treatment safety with no serious adverse events and 53% of continent patients showed significant improvement in erectile function at 12 months (48).

Sabrina T. Hansen evaluated the safety and preliminary efficacy of a single intracavernosal injection (ICI) of autologous ADRCs for treating ED following radical prostatectomy. The findings indicated that ADRCs may improve erectile function in the short term, particularly in patients without urinary incontinence (49).

However, there were currently no clinical studies on ADRCs treatment for DMED, which may represent a major future research direction.

### ADSCs exosomes

ADSCs secrete nano-sized membrane vesicles (40–100 nm in diameter) that carry functional proteins, mRNAs, microRNAs and tRNAs. These vesicles participated in intercellular communication through paracrine signaling and exhibited numerous biological functions (50).

A study by Fengzhi Chen had demonstrated for the first time that exosomes secreted by ADSCs could significantly improve erectile function in type 2 diabetic rats by inhibiting apoptosis of corpus cavernosum endothelial cells and smooth muscle cells. The therapeutic effect was comparable to direct ADSC injection (35).

M Li found that ADSC-Exosomes significantly increased the mean intracavernous pressure/mean arterial pressure (ICP/MAP) ratio in the bilateral cavernous nerve injury (BCNI) rat model, improving erectile function. The exosomes functioned through a paracrine mechanism, offering a potential therapeutic strategy for post-radical prostatectomy ED (51).

### Genetically modified ADSCs

Gene modification methods had been widely used to enhance the therapeutic efficacy of stem cells. The principle involved implanting ADSCs carrying the target gene into the body to regulate its expression, thereby improving the effectiveness of ED treatment.

Taotao Sun utilized the CRISPRa system to overexpress the relaxin family peptide receptor 1 (RXFP1) in ADSCs, significantly improving erectile function in DMED rats (52).

Jun Zhou’s research demonstrated that knocking down miR-423-5p in ADSCs significantly upregulated the expression of endothelial nitric oxide synthase (eNOS) and vascular endothelial growth factor A (VEGFA). In a DMED rat model, injection of modified ADSCs markedly improved erectile function (53).

Chao Luo’s study investigated the therapeutic effects and mechanisms of enhancing ADSC by downregulating NOD-like receptor protein 3 (NLRP3) in a DMED rat model. The results demonstrated that ASCsLV-shNLRP3 significantly improved endothelial function and smooth muscle repair, outperforming unmodified ADSCs (54).

The study by J Yang found that siPDE5-ADSCs transduced with a lentiviral vector significantly enhanced the secretion of insulin-like growth factor 1 (IGF-1) and VEGF, thereby accelerating the recovery of erectile function and corpus cavernosum structure in DMED rats (55).

Future research could employ bioinformatics methods to identify more ED-related genes and achieve their overexpression or knockdown in ADSCs, thereby enhancing the therapeutic efficacy of ADSCs for ED.

### Combination therapy

ADSCs could be combined with other treatment methods to enhance the therapeutic efficacy for ED.

The study by Shiyun Liu et al. demonstrated that the combination of Low-Intensity Pulsed Ultrasound(LIPUS) and ADSCs transplantation significantly improved erectile function in rats, promoted the secretion of angiogenic factors (such as VEGF) and enhanced corpus cavernosum endothelial function by activating Piezo ion channels and the ERK signaling pathway. This synergistic therapy offered a novel treatment strategy for DMED (56).

Xiyou Wang found that the combined use of ADSCs and the traditional Chinese medicine icariin significantly improved erectile function metrics (such as ICP/MAP) and enhanced the survival rate of transplanted ADSCs (57).

## Conclusion

Current research indicated that ADSCs demonstrated clear therapeutic efficacy in the treatment of DMED by improving vascular endothelial function, suppressing fibrosis and promoting nerve repair. However, their clinical translation still faced the following challenges: (1) optimal treatment parameters (cell dosage, administration frequency) have not yet been standardized; (2) long-term safety evidence remains limited; (3) the spatiotemporal dynamics of their mechanisms of action required further elucidation. Future efforts should focus on defining treatment protocols through multicenter randomized controlled trials and establishing interdisciplinary research frameworks to explore the synergistic effects of ADSCs with other therapies.
